# Histologic and functional outcomes of small intestine submucosa-regenerated bladder tissue

**DOI:** 10.1186/1471-2490-14-69

**Published:** 2014-08-23

**Authors:** Yiming Wang, Limin Liao

**Affiliations:** 1 Department of Urology, China Rehabilitation Research Center, 10 Jiaomen Beilu, Beijing 100068, Fentai District, China; 2 Department of Urology of Capital Medical University, Center of Neural Injury and Repair, Beijing Institute for Brain Disorders, 10 Youanmenwai Xitoutiao, Beijing 100069, Fentai District, China

**Keywords:** Small intestine submucosa, Regenerated bladder, Bladder augmentation, Urodynamics

## Abstract

**Background:**

Intestinal bladder augmentation has more disadvantages. One of the most promising alternative methods is tissue engineering in combination with surgical construction. Small intestine submucosa (SIS) is commonly used materials in tissue engineer. The aim of this study is determine the histologic and functional characteristics of SIS as bladder wall replacement in a rabbit augmentation model.

**Methods:**

18 New Zealand adult male rabbits, weight 2.5 ± 0.5Kg, were used in this study. The rabbits were divided into 3 groups of 6 based on the number of days post-operative (A, 4 weeks; B, 12 weeks; C, 24 weeks). All of the animals underwent urodynamic testing under anesthesia before cystoplasty with SIS patch. The cystometrograms were repeated 4, 12, and 24 weeks after surgery with the same method. SIS-regenerated bladder strips (10 × 3 × 3 mm) and normal bladder strips (10 × 3 × 3 mm) from the same bladder were obtained at 4, 12, and 24 weeks for in vitro detrusor strip study. The frequency and amplitude of the strip over 15 min was recorded. The regenerated tissue and normal tissue underwent histologic and immunocytochemical analysis. The results were quantified as optical density (OD) values.

**Results:**

Histologically, the SIS-regenerated bladders of group C (24 weeks post-operation) resembled normal bladder in that all 3 layers (mucosa with submucosa, smooth muscle, and serosa) were present. In the in vitro detrusor strip study, there were no significant differences in autorhythmicity and contractility between regenerated and normal tissues in group C (p > 0.05). Immunohistochemical analysis indicated that the quantity of A-actin grew to a normal level. Urodynamic testing showed that compliance remained stable in all groups post-operatively, and the volume increased 24 weeks post-operatively.

**Conclusion:**

Regenerated tissue has similar histologic and functional characteristics. SIS seems to be a viable material in the reconstruction of the rabbit urinary bladder.

## Background

Bladder augmentation is used to reduce the high bladder pressure that develops in patients with neurogenic bladder to protect the upper urinary system [1]. Gastrointestinal segment augmentation cystoplasty is associated with a number of complications, such as mucus production, stone formation, leakage and rupture, fibrosis, electrolyte imbalance, and development of bowel obstruction. [2,3]. All of these complications, which adversely affect the quality of life, give patients pause when offered a renal rescue operation. In view of these disadvantages, alternative patches have been investigated [4]. One of the most promising alternative methods is tissue engineering in combination with surgical construction [5-7].

Small intestine submucosa (SIS) is an acellular, non-immunogenic, biodegradable, xenogeneic, collagen-based material that is derived from the submucosal layer of porcine small intestine [8]. SIS has demonstrated regenerative capacities in multiple organ systems, including the aorta, vena cava, ligaments, tendons, abdominal wall, and skin [9,10]. The aim of our study was to determine the functional and histologic characteristics of SIS (Cook-SIS Technology, [Indiana, USA]) as bladder wall replacement in a rabbit augmentation model.

## Methods

**Figure 1:**
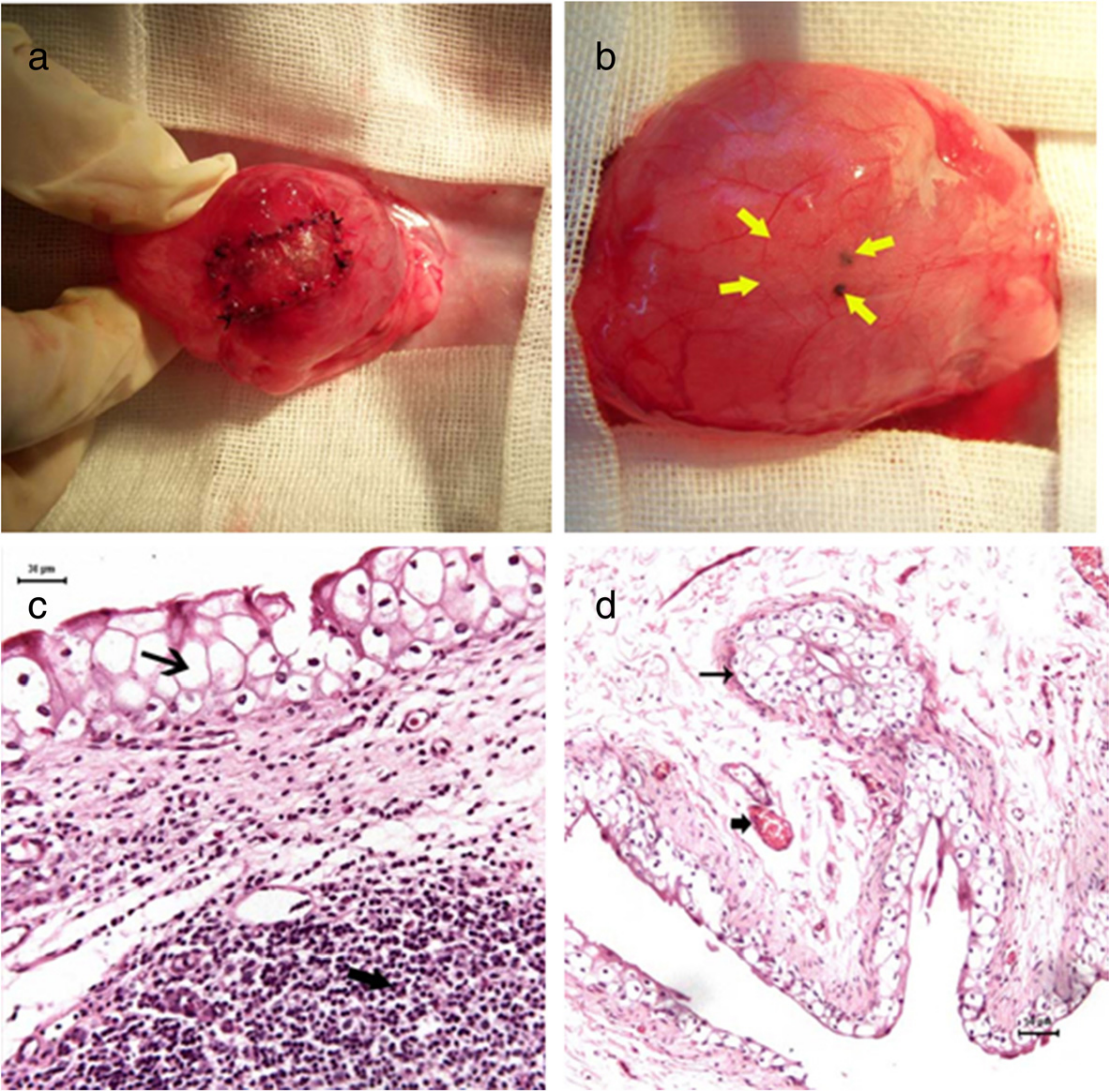
**Macroscopic and HE evaluation. a**. The SIS patch (1.0 × 2.0 cm) was already grafted onto the host bladder. **b**. Regenerated tissue in the arrows 24 weeks post-operation. **c**. Thin arrow marked the regenerated transitional epithelium in the region of the SIS graft. Coarse arrow marked the infiltrated inflammatory cells (×20). **d**. Thin arrow marked the regenerated transitional epithelium. Coarse arrow marked the new vessels (×10).

After approval of the ethics committee of the China Rehabilitation Research Center, 18 New Zealand adult male rabbits, weight 2.5 ± 0.5 Kg, were used in this study. All the animals were in good health. The rabbits were divided into 3 groups of 6 based on the number of days post-operative (A, 4 weeks; B, 12 weeks; C, 24 weeks). The abdominal regions of the rabbits were shaved after anesthesia with 2% phenobarbital (30 mg/kg). The abdomen was opened through a midline incision and the bladder was exposed. The anterior wall of the bladder was opened longitudinally through a 3-cm incision in the midline of the bladder body. The SIS patch (1.0 × 2.0 cm) was grafted onto the host bladder with a 5/0 vicryl interrupted suture (Figure 1a). Four 5/0 silk marking stitches were placed outside the bladder wall near the corners of the patch. Perivesical fat was fixed over the bladder wall to cover the graft and the abdominal wall was closed anatomically. A single dose of ceftriaxone was administered (500 mg intramuscular). Neither urinary diversion nor urethral catheterization was used. The rabbits were housed and fed in separate cages.

### Urodynamic test

All of the animals underwent urodynamic testing under anesthesia with 2% Phenobarbital (30 mg/kg) before cystoplasty. Cystometrograms (CMGs) were carried out with a 5 F double-lumen urodynamic catheter placed through the urethra and a continuous infusion (10 ml/min) of sterile saline via the catheter. The saline infusion was stopped at the first sign of overflow incontinence. Then the CMGs were repeated twice, the averaged maximal capacity was recorded. Bladder compliance was calculated as the change in volume divided by the change in pressure. The CMGs were repeated 4, 12, and 24 weeks after surgery with the same method. Data are expressed as the mean ± standard error of the mean.

### In vitro detrusor strip study

The entire bladder was removed post-operatively after CMGs at 4, 12, and 24 weeks and placed in Krebs solution continually bubbled with 95% O_2_ and 5% CO_2_. This solution contained the following compounds in g/L: NaCl, 6.92; KCl, 0.35; KH_2_PO_4_, 0.16; MgSO_4_.7H_2_O, 0.29; NaHCO_3_, 2.1; CaCl_2_, 0.35; and glucose, 2.0. SIS-regenerated bladder strips (10 × 3 × 3 mm) and normal bladder strips (10 × 3 × 3 mm) from the same bladder were obtained. Silk ligatures were used to mount the strips vertically between two suspension clasps in organ bath chambers. The tissue was continually immersed in Krebs solution at 37°C. Tension on the strips was measured using a force-displacement transducer. Tension (1 g) was applied to the strips. The frequency and amplitude of the strip over 15 min was recorded. Data are expressed as the mean ± standard error of the mean.

### Histologic and immunocytochemical analyses

As controls for each rabbit, a full thickness bladder fragment was excised distal from the grafted area. Formalin-fixed specimens from the grafted and normal areas of the bladder were embedded in paraffin. Five μm sections were cut and stained with hematoxylin and eosin (HE). A-actin (BIOSS Inc., Massachusetts, USA) was also used for immunohistochemical evaluation of smooth muscle regeneration. The main steps of immunohistochemistry methods:(1) Put the slide with paraffin section in drying oven 2 hours, 60°C. (2) Put it in xylene 15 min, 3 times. (3) Then in ethanol, from 100% to 95%, then 90%, 80%, 70%, each 5 min. (4) Wash with water one time, 5 min, then transform into PBS (0.01 M, pH 7.4), wash 5 min, 3 times. (5) Antigen retrieval: put the slide into citrate buffer (0.01 M, pH 6.0), keep the solution in boiling water for 10-15 min, cool down to room temperature (6) Wash with PBS (0.01 M, pH 7.4) 5 min, 3 times (7) Block endogenous peroxidase by 3% H2O2 for 30 min (8) Wash with PBS (0.01 M, pH 7.4) 5 min, 3 times (9) Incubation with blocking buffer (normal goat serum or 3% BSA) at 37°C for 20 min (10) Discarding the goat serum and droping the primary antibody A-actin (BIOSS Inc, USA) with diluted in PBS (0.01 M PBS, pH 7.4,1:100–500), incubating the sections overnight at 4°C (11) Wash with PBS (3X5 min) (12) Add secondary antibody, Goat Anti-rabbit IgG,(BIOSS Inc, USA) incubating the sections for 20 min at 37°C (13) Wash with PBS (3X5 min) (14) Add Avidin/HRP, incubating the sections for 20 min at 37°C (15) Wash with PBS (3X5 min) (16) Colouration with 3,3-diaminobenzidin (DAB), Observe by microscope, stop colouration with the distilled water at the right time (17) Dehydration in ethanol, from 70% to 80%, then 90%, 95%, 100%, each 5 min, then transform into xylene 10 min, 3 times (18) Cover with coverslip by neutral gums. The preparations were observed under a light microscope. For the convenience of analysis, the results were quantified as optical density (OD) values. Using analysis software (Leica Qwin, v2.3; Leica Inc. Solms, Germany) with the same parameters (exposure time, 22 ms; light intensity, 60%), the mean OD of A-actin was measured.

### Statistical analysis

In this study, self-control was approached. Quantitative data were compared using t-tests. All statistical analyses used SPSS 13.0 (SPSS, Inc., Chicago, IL, USA).

## Results

### Macroscopic evaluation

**Figure 2:**
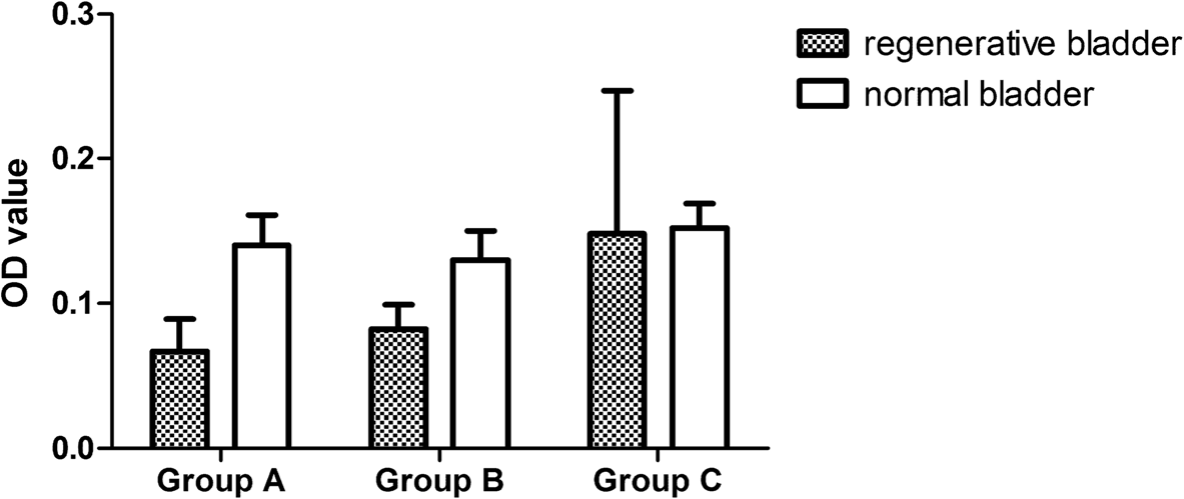
**Compare of OD value.** The OD value of regenerated tissue was 0.067 ± 0.022 in group A, 0.082 ± 0.017 in group B, and 0.148 ± 0.099 in group C. Compared with normal bladder (0.140 ± 0.021 in group A, 0.130 ± 0.020 in group B, 0.152 ± 0.017 in group C), groups A and B were significantly different with normal bladder (p < 0.05), while there was no significant difference in group C and normal bladder (p > 0.05).

All rabbits were euthanized. The outer and inner surfaces of the bladder, perivesical fat, kidneys, and ureters were evaluated macroscopically. The kidneys and ureters were grossly normal without evidence of hydronephrosis. There were no diverticula in any group. There was no extravasation or contractions in the graft regions. The material was degraded in groups B and C, but not degraded on the inner surface in group A. Regenerated tissue covered the outer surface of the region of the graft, which was indistinguishable from the normal host bladder at the outer and inner surfaces in group C (Figure 2b).

### Microscopic evaluation

Continuation of host transitional epithelium in the region of the SIS graft was observed under light microscopy in all groups. Inflammatory cells infiltrated regenerated epithelium 4 weeks after surgery (group A; Figure 1c), and nearly disappeared 12 weeks post-operatively (group B). Detrusor was formed 12 weeks post-operatively (group B). These layers were indistinguishable from normal bladder 24 weeks post-operatively (group C).

### Immunohistochemical evaluation

Detrusor was present and well-visualized with A-actin staining. The detrusor component was scarce in group A, while the detrusor fiber was more evident in group B than in group A, but smaller in number and size compared with normal tissue (P = 0.001). There was no significant difference in the OD value of A-actin staining in group C and normal bladder (P = 0.759) (Figure 2).

### Urodynamic test

Pre-operatively, the bladder capacities in groups A, B, and C were 40.30 ± 7.6, 55.7 ± 27.2, and 48.98 ± 27.8 ml, respectively. Post-operatively, the capacities were 26.11 ± 4.8, 60.4 ± 24.7, and 57.80 ± 27.7 ml, respectively. There was a significant decrease in the bladder volume of group A, and no significant difference in group B; however, the bladder volume was significantly increased in group C (24 weeks post-operation; P = 0.001). The bladder compliance of groups A, B, and C pre-operatively was not significantly different post-operation (Table 1).

**Table 1:** Comparisons between regenerated and normal bladder and results of urodynamic testing

	**Group A**		**Group B**		**Group C**	
	**Pre-op**	**Post-op**	**Pre-op**	**Post-op**	**Pre-op**	**Post-op**
Bladder volume (ml)	40.30 ± 7.6*	26.11 ± 4.8*	55.7 ± 27.2	60.4 ± 24.7	48.98 ± 27.8*	57.80 ± 27.7*
	P = 0.021		P = 0.111		P = 0.001	
Compliance (ml/cmH_2_O)	2.74 ± 1.57	2.36 ± 0.71	4.9 ± 2.04	5.1 ± 1.54	6.88 ± 4.07	6.2±3.86
	P = 0.653		P = 0.272		P = 0.732	

*P < 0.05 was considered statistically significant.

### In vitro detrusor strip study

Regenerated detrusor strips in group A had visible slight vibration waves, the frequency and amplitude of which could not be measured; the group A strips were significantly different from normal detrusor strips. In group B, the frequency (min-1) of regenerated detrusor strips was 2.88 ± 0.49 (min-1) and the amplitude was 0.13 ± 0.014 (g), which was significantly different from normal detrusor strips. The frequency (3.64 ± 0.98) and amplitude (4.35 ± 1.25) of regenerated detrusor strips in group C were not significantly different compared with normal detrusor strips (Table 2).

**Table 2:** Comparisons between regenerated and normal bladder tissues and the results of the detrusor strip study

	**Group A**		**Group B**		**Group C**	
	**Normal**	**Regenerated**	**Normal**	**Regenerated**	**Normal**	**Regenerated**
Frequency (min^−1^)	___		4.80 ± 1.20*	2.88 ± 0.49*	3.64 ± 0.98	4.35 ± 1.25
			(p = 0.005)		(P = 0.442)	
Amplitude (g)	___		0.47 ± 0.083*	0.13 ± 0.014*	0.67 ± 0.09	0.50 ± 0.25
			(p = 0.000)		(p = 0.051.)	

*P < 0.05 was considered statistically significant.

## Discussion

To create a target volume and functional regenerated bladder are challenges in tissue engineering. We chose SIS as materials used for bladder regeneration; using SIS with the help of host tissues gives successful results [6]. In recent years, bladder regeneration using SIS has been reported in rat and dog models [11,12]; however, research involving functional bladder regeneration of rabbits has seldom been reported. We showed that regenerated bladder tissue has similar function in vitro strip studies with normal tissue and as part of new bladder post-operation compared with pre-operative bladder.

We chose male rabbits as our study animals because the external urethral orifice of male rabbits is easier to identify than the urethra in female rabbits. Urodynamic testing was carried out after opening the abdomen under anesthesia to remove the influence of abdominal pressure. We did not choose cystometry via cystostomy as has been previously reported [13]. We attempted to maintain the complete bladder, as excessive damage to the bladder tissue might adversely impact the results. The bladder volume of group A (26.11 ± 4.8 ml) was decreased significantly compared with the pre-operative volume (P = 0.021). Encrustration of the bladder was observed all cases in group A (4 weeks post-operation). It has been suggested that encrustration is a result of high urate levels in rabbits [14]. And the encrustration all disappeared at last [15]. We suggest that encrustation might be the patch which was not degraded because encrustation was not noted in group B or C. Furthermore, we found that in group A the region of the patch surrounded by a large area of inflammatory polyp hyperplasia. All of these observations could be the result of decreased bladder capacity in group A.

The bladder volume was significantly increased 24 weeks post-operation (group C; P = 0.001). In the current study, the reduction in bladder capacity was transient and the bladder capacity was eventually expanded.

Furthermore, the results showed that compliance post-operation was similar with compliance pre-operation in group C (p = 0.051). The regenerated bladder tissue did not reduce the original normal bladder compliance. Moreover, in the in vitro detrusor strip study, regenerated tissue had a slight contraction wave in group A. Based on HE staining, group A showed scattered *de novo* tissue formation of muscle fibers, thus muscle regeneration began to emerge in the patch area 4 weeks post-operation, albeit a small number and discontinuous. Therefore, a smaller contraction wave was recorded in group A. The regenerated strips were similar to normal muscle strips with a spontaneous contraction frequency and amplitude in group C, which were not statistically different (frequency, p = 0.442; amplitude, p = 0.051). Regenerated smooth muscle bundles were also observed in regenerated tissue by HE staining in which the arrangement was also similar to normal tissue with a vertical outer ring-type arrangement.

Sandusky et al. [16] has offered two explanations regarding the smooth muscle regeneration process: (1) normal smooth muscle tissue growth from the patch edge inward; and (2) *de novo* smooth muscle is derived from peripheral cells, which includes the epithelium of capillaries. The author believes that both explanations might exist, but further trials are needed. SIS may also promote muscle regeneration. Many studies have identified the structure, and demonstrated the useful biological properties in tissue regeneration since discovery in 1987 [6,13]. Based on the results of urodynamic testing and the in vitro detrusor strip study, we conclude that along with SIS functional bladder was regenerated.

Some studies [17] have reported that the patch area is infiltrated by a large number of fibroblasts and inflammatory cell, 1–4 weeks after transport. These cells secrete all types of materials, such as TGF-β, that can determine tissue regeneration.

Actin is one of the primary contractile proteins in bladder smooth muscle cells. Currently, A-actin is considered to be the main phenotype indicator in contractile smooth muscle cells [18]. Immunohistochemical studies have shown that A-actin stains brown. The OD was used for statistical analysis; higher OD values indicated increased expression of A-actin. The OD value increased with time post-operation. The OD value reached a level similar to normal tissue in group C (24 weeks post-operation).

## Conclusion

We conclude that histologic and functional regeneration of bladder in rabbit can be achieved with SIS. Therefore, SIS seems to be a viable material in the reconstruction of the rabbit urinary bladder. Advanced and more detailed researches on whether or not regeneration of normal tissue can be accomplished from a pathologic organ should be carried out.

## Competing interests

The authors declare that they have no competing interests.

## Authors’ contributions

YW performed the animal experiments and drafted the manuscript. LL contributed to the conception and design of the study, analysis interpretation of data, and helped to draft the manuscript. Both authors read and approved the final manuscript.

## Authors’ information

YW is MD and urologist in the department of urology of China Rehabilitation Research Center (CRRC). LL, MD & PhD, is chairman of the department of urology of CRRC, and a professor of urology and vice-chairman of urologic department of Capital Medical University in Beijing. His main interests are neurourology, urodynamics and incontinence. He is a committee member of the neurourology promotion committee of the international continence society (ICS), and was chairman of 42nd ICS annual meeting in Beijing.

Yiming Wang and Limin Liao are co-first authors.

## Pre-publication history

The pre-publication history for this paper can be accessed here:

http://www.biomedcentral.com/1471-2490/14/69/prepub
